# Metal-free organic dyes for TiO_2_ and ZnO dye-sensitized solar cells

**DOI:** 10.1038/srep18756

**Published:** 2016-01-07

**Authors:** Gurpreet Singh Selopal, Hui-Ping Wu, Jianfeng Lu, Yu-Cheng Chang, Mingkui Wang, Alberto Vomiero, Isabella Concina, Eric Wei-Guang Diau

**Affiliations:** 1 SENSOR Lab, Department of Information Engineering, University of Brescia, Via Valotti 9, 25133 Brescia, Italy; 2 CNR-INO SENSOR Lab, Via Branze 45, 25123 Brescia, Italy; 3 Department of Applied Chemistry and Institute of Molecular Science, National Chiao Tung University, Hsinchu 30010, Taiwan; 4 Michael Grätzel Center for Mesoscopic Solar Cells, Wuhan National Laboratory for Optoelectronics, School of Optical and Electronic Information, Huazhong University of Science and Technology, 1037 Luoyu Road, Wuhan 430074 (P. R. China); 5 Luleå University of Technology, 971 98 Luleå, Sweden

## Abstract

We report the synthesis and characterization of new metal-free organic dyes (namely B18, BTD-R, and CPTD-R) which designed with D-π-A concept to extending the light absorption region by strong conjugation group of π-linker part and applied as light harvester in dye sensitized solar cells (DSSCs). We compared the photovoltaic performance of these dyes in two different photoanodes: a standard TiO_2_ mesoporous photoanode and a ZnO photoanode composed of hierarchically assembled nanostructures. The results demonstrated that B18 dye has better photovoltaic properties compared to other two dyes (BTD-R and CPTD-R) and each dye has higher current density (*J*_sc_) when applied to hierarchical ZnO nanocrystallites than the standard TiO_2_ mesoporous film. Transient photocurrent and photovoltage decay measurements (TCD/TVD) were applied to systematically study the charge transport and recombination kinetics in these devices, showing the electron life time (*τ*_R_) of B18 dye in ZnO and TiO_2_ based DSSCs is higher than CPTD-R and BTD-R based DSSCs, which is consistent with the photovoltaic performances. The conversion efficiency in ZnO based DSSCs can be further boosted by 35%, when a compact ZnO blocking layer (BL) is applied to inhibit electron back reaction.

Dye sensitized solar cells (DSSCs)^1^ developed by O’Regan and Grätzel, have attracted considerable attention since 1991, promising to be among the most interesting alternatives to conventional solid–state semiconductor solar cells, being in principle cheap, environmentally compatible and large-area scalable. A typical DSSC is composed of a mesoporous nanostructured titanium oxide thin film, whose surface is covered with a monolayer of dye molecules, a redox -couple electrolyte and a platinized fluorine-doped tin oxide (FTO) glass as counter electrode. For many years, ruthenium polypyridyl complexes were successfully applied as light harvesters, yielding overall conversion efficiency (*η*) more than 11% under AM 1.5 simulated sunlight (100 mWcm^−2^)^2^. Recently, this *η* set a new record value exceeding 14% by using porphyrin dye molecule together with cobalt-based redox couple electrolyte^3^,^4^. Ruthenium complexes are however still the most commonly studied sensitizers for DSSCs, although they are unsuitable for large scale commercialization due to their high cost and scarce availability of ruthenium. To overcome this problem, strong research efforts have been addressed to synthesize ruthenium-free dyes. Metal-free organic dyes have attracted great interest in this respect due to several advantages they offer: (i) high molar extinction coefficients; (ii) easily tunable opto-electronic properties; (iii) enhanced environmental compatibility and abundance of their constituents; (iv) reduced production costs^5^. The highest *η* has been achieved with mesoporous TiO_2_ nanocrystallites film sensitized by different metal-free organic dye molecules: hemicyanine dye (*η* = 5.1%)^6^, thienylfluorene dye (*η* = 5.23%)^7^, phenothiazine dye (*η* = 5.5%)^8^, thienothiophene-thiophenederived dye (*η* = 6.23%)^9^, N, N-dimethyl-anilinecyanoacetic acid (*η* = 6.8%)^10^, porphyrin dye (*η* = 7.1%)^11^, modified D-π-A dye (DEK1, *η* = 7.17%)^12^, oligothiophene dye (*η* = 7.7%)^13^, coumarin dye (*η* = 8.2%)^14^, oligo-phenylene vinylene-unit dye (*η* = 9.1%)^15^ and indoline dye (D205, *η* = 9.52%)^16^. Recently Demadrille *et al*. reported new metal free organic dye (RK1) with a photoconversion efficiency as high as 10.2%, which is comparable to N719 dye^17^.

In metal-free organic dye a donor-acceptor couple, D-π-A, is present, in which the electron donor is not only able to tune the electronic coupling with the acceptor, but also determines the molecule adsorbed state on the titania or zinc oxide nanocrystal in DSSC devices. Several investigations about the donor modification were reported in recent years; in particular, the organic dye D35^18^ and derivatives (Y123^19^, LEG4^20^), featuring phenyl extended triphenyl amine donor, gained popularity because of the prominent performance in DSSC devices. It was reported that, when an extended triphenyl amine is used as the donor group, an up-shift of TiO_2_ conduction band (CB) edge (CBE) can be observed on account of the increased net surface dipole moment of the adsorbed dye molecules.

On the other hand, by inserting specific aromatic units into the π bridge, one can broaden the light-harvesting region of dyes from visible to near-IR region. In this case, several highly efficient dyes were obtained, including the popular LEG-series^21^, push-pull porphyrins^22^ and the D-A-π-A WS-series dyes^23^. Inspired by their pioneer work, we designed and synthesized a series of novel organic dye, namely: (E)-2-(5-((7-(4-(bis(4-(5-(4-(hexyloxy)phenyl)thiophen-2-yl)phenyl)amino)phenyl)-2,3-dihydrothieno[3,4-b][1,4]dioxin-5-yl)methylene)-4-oxo-2-thioxothiazolidin-3-yl)acetic acid (labeled as B18), (E)-2-(5-((6-(4-(bis(4-(5-(4-(hexyloxy)phenyl)thiophen-2-yl)phenyl)amino)phenyl)-4,4-didodecyl-4H-cyclopenta[1,2-b:5,4-b’]dithiophen-2-yl)methylene)-4-oxo-2-thioxotetrahydrothiophen-3-yl)acetic acid (labeled as CPTD-R) and (E)-2-(5-((5-(7-(4-(bis(4-(5-(4-(hexyloxy)phenyl)thiophen-2-yl)phenyl)amino)phenyl)benzo[c] [1,2,5]thiadiazol-4-yl)thiophen-2-yl)methylene)-4-oxo-2-thioxotetrahydrothiophen-3-yl)acetic acid (labeled as BTD-R) featuring with 2-(4-(hexyloxy) phenyl) thiophene extended donor, and EDOT, CPDT, and BTD-thiophene π bridge.

Concerning the electron transport material, intrinsic transport limitations affect TiO_2_, thus limiting the possibilities of enhancing the photoconversion efficiency of DSSCs exploiting this metal oxide as photoanode^24^,^25^,^26^,^27^. Other *n*-type metal oxide semiconductors, such for instance ZnO, SnO_2_, Nb_2_O_5_ and In_2_O_3_ can be used as alternative photoanode materials^28^,^29^,^30^,^31^,^32^,^33^,^34^,^35^,^36^, as well as composite systems, e.g. carbon nanotubes^37^,^38^ or graphene^39^ mixed with TiO_2_ nanoparticles. Among all, ZnO is very promising, due to its electronic band structure (very similar to TiO_2_)^30^ and high electron mobility (one order higher than TiO_2_)^40^,^41^,^42^. In a very recent paper^43^, Grätzel and co-workers compared ZnO- and TiO_2_-DSSCs composed of large insulating Al_2_O_3_ particles covered with thin layers of either ZnO or TiO_2_. They pointed out that the performances of the two kinds of cells are similar. However, the higher photogenerated electron transport rate contributes to cell performance for ZnO, while in TiO_2_ a low recombination rate, combined with higher dye loading and faster electron injection boost *η*. Due to its large availability and uncountable obtainable low dimension structures, such as nanorods/nanowires^20^,^44^, nanotubes^45^,^46^, nanosheets^47^,^48^, nanoflowers^49^, tetrapods^50^,^51^,^52^ and hierarchical aggregates^53^,^54^,^55^, ZnO is a very interesting alternative to TiO_2_ to investigate. Application of these nanostructured ZnO photoanodes in DSSC significantly enhanced *η*, compared to simple ZnO nanoparticle mesoporous films. To the best of our knowledge, the highest *η* (7.5%) was reported by hierarchical assembled ZnO nanocrystallites^53^, which offer large specific surface area for dye loading while poly-dispersed aggregates act as efficient light scatterers, enhancing the probability of photon absorption. Another strategy to boost *η* in ZnO DSSCs is the application of a ZnO buffer layer (BL) in between the FTO and the ZnO active layer, to inhibit the electron back reaction from the FTO to electrolyte and to enhance the chemical capacitance during the transport and collection processes^56^,^57^,^58^.

Herein we present the details of the synthesis of three new metal-free organic dyes and apply them as light harvesters in both TiO_2_- and ZnO-based DSSCs. A systematic comparison of the photovoltaic properties of these dyes is carried out on (i) standard TiO_2_ mesoporous films and (ii) spray deposited hierarchical ZnO nanocrystallites in order to highlights the effect of photoanode materials and we found that all dye molecules outperform in term of current density (*J*_sc_) with hierarchical ZnO nanocrystallites than standard TiO_2_ mesoporous films. In addition, the effect of BL on the functional performance of ZnO-based DSSCs is also investigated. Charge transport and recombination kinetics in these devices are evaluated by applying transient photocurrent and photovoltage decay (TCD/TVD). The results of this work can contribute to the development of ZnO-based DSSCs by properly designing metal-free organic dyes, which are typically optimized for TiO_2_, limiting the potential of ZnO photoanodes.

## Results and Discussion

### Dye molecules (B18, BTD-R and CPTD-R) characterization

**Figure 1:**
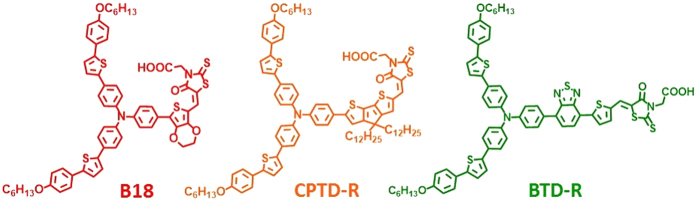
Molecular structures of the B18, CPTD-R and BTD-R organic dyes.

**Figure 2:**
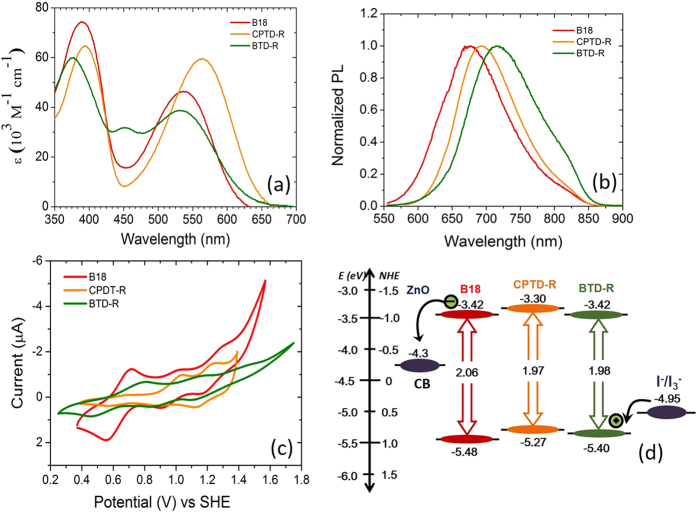
Characterization of dye molecules: (**a**) absorption and (**b**) emission spectra of B18, CPTD-R and BTD-R in CHCl_3_ solution. (**c**) Cyclic voltammograms of B18, CPTD-R and BTD-R, obtained in in freshly distilled CHCl_3_ using 0.5 M Tetrabutylammonium hexafluorophosphate (TBAPF_6_) as supporting electrolyte in a three–electrode configuration. (**d**) Schematic of potential levels of B18, CPTD-R and BTD-R with HOMO and LUMO levels showing electron injection to CB of ZnO and dye regeneration by I^−^/I_3_^−^ electrolyte.

Synthetic procedures used for the preparation of B18, CPRD-R and BTD-R dyes are reported in Supporting Information and the structures of dyes are reported in Fig. 1. The dyes contain a rhodanine-3-acetic acid as anchoring group and triphenyl amine as the donor centrum. Figure 2 shows a systematic comparison of molecule (B18, BTD-R and CPTD-R) optical and electrochemical characterizations and corresponding values are summarized in Table 1. Figure 2 (a) shows the absorption spectra of the dye molecules in CHCl_3_ solution. All molecules feature absorption in a rather broad range, between 350 and 650 nm, which is a typical characteristic of the π-conjugated donor-acceptor type chemical architecture. The longer wavelength absorption band is mainly due to the π-π* charge transfer transitions from the donor to the cyanoacrylic acid acceptor of the dye molecule. B18, CPDT-R and BTD-R molecules exhibit absorption peaks in the visible region of the spectrum at 537, 565 and 530 nm respectively. The significant red-shift of the absorption peak from 537 to 565 nm in case of CPTD-R with respect to B18 dye is due to presence of a cyclopentadithiopene (CPDT) unit as the π−conjugation. BTD-R molecule, due to the presence of a D-A-π–A configuration, shows a much broader absorption band from 500 to 650 nm compared to B18 and CPTD-R. Moreover, very good extinction coefficients, in the order of 10^4^ M^−1^ cm^−1^, are identified for all dyes.

Emission spectra of dye molecules are shown in Fig. 2(b) and corresponding data are listed in Table 1. Fluorescence spectrum of B18 exhibits a broad emission peak at 677 nm, while CPDT-R and BTD-R feature the emission maximum at 694 nm and 715 nm, respectively. This shift of the maximum fluorescence emission to longer wavelengths is observed due to the introduction of D-A-π–A configuration for BTD-R. We employed cyclic voltammetry (CV) to investigate the reduction potentials of the organic dyes (Fig. 2(c) and Table 1). All dyes exhibit reversible oxidation curves, which are ascribed to the removal of an electron from the amine segment. The first oxidation potentials (E_ox_) of the dyes correspond to the highest occupied molecular orbital (HOMO) energy. Reduction potentials are obtained from the equation E_red_ = E_ox_–E_0–0_, in which E_0–0_ is the zero–zero excitation energy obtained from the intersection of absorption and emission spectra at the absorption edge. Energy alignment of the organic dye molecules with the device components can be obtained, which is presented in Fig. 2(d), along with the position of the conduction band (CB) of ZnO and the standard redox potential of the electrolyte used in devices (I^−^/I_3_^−^). Figure 2(d) shows the lowest unoccupied molecular orbital (LUMO) levels of B18, CPDT-R and BTD-R, positioned at −3.42, −3.30, and −3.42 eV, respectively, which are higher than the CB of ZnO (−4.3 eV). The driving forces for hot-electron injection are thus 0.88, 1.0, and 0.88 eV, respectively. On the other hand, the HOMO levels are located at −5.48, −5.27, and −5.40 eV, and the driving forces for oxidized dye regeneration reaction are 0.53, 0.32, and 0.45 eV, which are also satisfying for the regeneration reaction by the applied electrolyte.

### Photovoltaic performance with standard mesoporous TiO_2_

**Figure 3:**
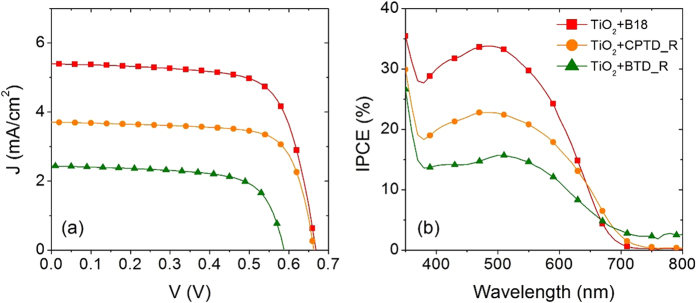
Photovoltaic properties of standard mesoporous TiO_2_ DSSCs sensitized with the three different metal free organic dyes. (**a**) Current density vs photovoltage curves under 1 sun illumination (AM 1.5 G, 100 mW cm^−2^); (**b**) IPCE spectra.

Figure 3(a) shows the comparison of current density *vs* photovoltage (*J*-*V*) characteristics of standard mesoporous TiO_2_ film sensitized by the three metal-free organic dyes. The corresponding photovoltaic parameters, namely short circuit current densities (*J*_sc_), open circuit voltages (*V*_oc_), fill factor (*FF*) and photoconversion efficiencies (*η*), are reported in Table 2. Photocurrent densities (*J*_sc_) and open circuit voltages (*V*_oc_) for these devices follow the trend B18 > CPTD-R > BTD-R. Since all the other components of the cells (photoanode, electrolyte and counter electrode) are the same, the difference in functional performances is mainly dependent on the properties of dye molecules such as: (i) electron injection efficiency (position of LUMO level with respect to the CB of semiconductor); (ii) oxidized dye regeneration efficiency (position of HOMO level with respect to standard redox potential of I^−^/I_3_^−^); (iii) light harvesting efficiency (γ_LHE_). To enhance device performances, dye molecules should have optimized electron injection, fast oxidized dye regeneration reaction and high γ_LHE_. Among all dyes molecules, CPTD-R dye molecule has higher potential for electron injection compared to other two dyes, whereas B18 has higher driving force for oxidized dye regeneration than CPTD-R and BTD-R.

The device sensitized with B18 dye shows better functional performances in term of *J*_sc_ and *V*_oc_, hence *η*, compared to devices exploiting CPTD-R and BTD-R as light harvesters, possibly due to the favorable combination of electron injection and good potential for oxidized dye regeneration. Short circuit photocurrent density, in particular, is enhanced when B18 is used as light harvesters (5.40 mA cm^−2^), being more than two times higher than that recorded for BTD-R (2.40 mA cm^−2^) and almost 30% higher that the *J*_sc_ obtained for CPTD-r (3.80 mA cm^−2^). A slight improvement in *V*_oc_ is observed by comparing devices working with B18 and CPTD-R (669 mV and 657 mV, respectively), while a significant difference in *V*_oc_ (79 mV) is revealed by analyzing cells sensitized with B18 and BTD-R (669 mV and 590 mV, respectively). Rather good FFs are recorded for all the analyzed devices (around 70%).

**Figure 4:**
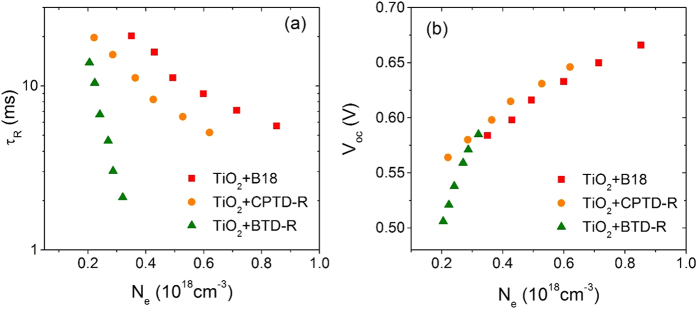
Comparison of electron-transport kinetics for three different dyes sensitized standard mesoporous TiO_2_: (**a**) *τ*_*R*_ vs *N*_*e*_; (**b**) *V*_*oc*_ vs *N*_*e*_ (*τ*_*R*_: electron lifetime, *V*_*oc*_: open-circuit voltage and *N*_e_: charge density).

As for the devices with CPTD-R and BTD-R dyes, CPTD-R dye features better photovoltaic performance than BTD-R. The possible reason behind this behaviour is the dominancy of electron injection process over the oxidized dye regeneration process, since CPTD-R has higher electron injection driving force than BTD-R (see Fig. 2(d)). These results are further confirmed by incident photon to current conversion efficiency (IPCE) and TCD/TVD analyses: better IPCE values (see Fig. 3(b)) and longer *τ*_*R*_ (Fig. 4(a)) were indeed observed for the device sensitized with B18 dye, compared to devices using CPTD-R and BTD-R dyes.

The IPCE of the device is a function of light harvesting efficiency of dye molecules (γ_LHE_), quantum yield of electron injection from the LUMO of the excited dye (γ_in_) and collection efficiency (γ_col_), as follows: IPCE = γ_LHE_ × γ_in_ × γ_col_^59^. IPCE spectra of three different TiO_2_-based DSSCs are displayed in Fig. 2(b). The highest value of IPCE is: 34% (at 488 nm) for B18, 25% (at 486 nm) for CPTD-R and 16% (at 515 nm) for BTD-R. In the wavelength range 400–600 nm, the IPCE follows the trend: B18 > CPTD-R > BTD-R, consistent with the trend in *J*_sc_ (Table 2). All devices are identical in all respect, but for the dye molecules, so differences in IPCE can be reasonably attributed to different dye molecule properties, especially γ_LHE_ and γ_in_.

In order to better elucidate the mentioned trend of functional performances, we applied transient current density decay (TCD) and transient voltage decay (TVD) analyses, reported in Fig. 4.

Figure 4(a) displays the plots of electron lifetime (*τ*_R_) versus charge density (*N*_e_) for TiO_2_ photoanodes sensitized with the three dye molecules at six different light intensities. At particular value of *N*_e_ (0.3 × 10^18^ cm^−3^), the *τ*_R_ follow the trend B18 > CPTD-R > BTD-R (see Table S2). This is consistent with the functional performances reported in Table 2, and suggest a reduced charge carrier recombination between the metal oxide CB and the electrolyte, reasonably associated to the observed higher *V*_oc_ value for the B18 and CPTD-R sensitized devices than BTD-R. No significant difference in TiO_2_ CBE position is observed (Fig. 4(b)), when compared at particular value of *N*_e_ (0.3 × 10^18^ cm^−3^) as reported in Table S2, which suggest charge carrier recombination is dominant in determining *V*_oc_ for devices, when sensitized with different light harvesters.

Overall, low functional performances are identified for TiO_2_-based DSSCs: investigation of the trend of functional parameters *vs* photoanode thickness (reported in Figure S4) indicates that, after reaching a maximum at around 11 μm, every functional parameter is decreasing. This is a typical behaviour for TiO_2_ standard photoanodes and suggest that observed performances are related to dye molecules, which seem not suitable to synergistically work with titanium dioxide to enhances device capability of converting solar energy.

### Photovoltaic performance with hierarchical ZnO nanocrystallites

Metal-free dye molecules were also applied as light harvesters in ZnO-based DSSCs, using hierarchical structured ZnO (Figure S1), whose detailed characterization can be found in ref. 53. Briefly, the active layer, composed of polydispersed aggregates with broad size distribution range (between 100 nm and 600 nm), is especially designed for optimization of light managing and charge transport, while maintaining large specific surface area for dye loading. A transparent and compact ZnO BL is deposited in between the FTO glass and the ZnO active layer, aimed at physically insulating the FTO from the electrolyte, thus reducing charge recombination at this interface^60^. The BL is composed of homogeneously distributed rough lamellae having thickness of a few tens of nanometers and lateral dimensions in the sub-micrometer range and are oriented normal to substrate plane.

**Figure 5:**
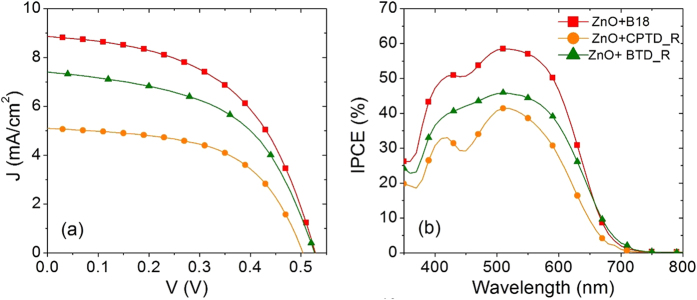
Photovoltaic properties. (**a**) Current density vs photovoltage curves under 1 sun illumination (AM 1.5 G, 100 mW cm^−2^); (**b**) IPCE spectra of three different metal free organic dyes sensitized hierarchical structured ZnO DSSCs.

Figure 5(a) and Table 2 present the systematic comparison of *J*-*V* characteristics of hierarchical ZnO nanocrystallites sensitized by the three different metal-free organic dyes. B18 dye shows the best functional performances also in the case of a ZnO-based electrode, ascribable in particular to a high photocurrent density (as high as 8.85 mA cm^−2^). No significant differences are observed in *V*_oc_ between devices sensitized with B18 and BTD-R dyes, while a slightly reduced *V*_oc_ is recorded for CPTD-R (Fig. 5 (a)).

**Figure 6:**
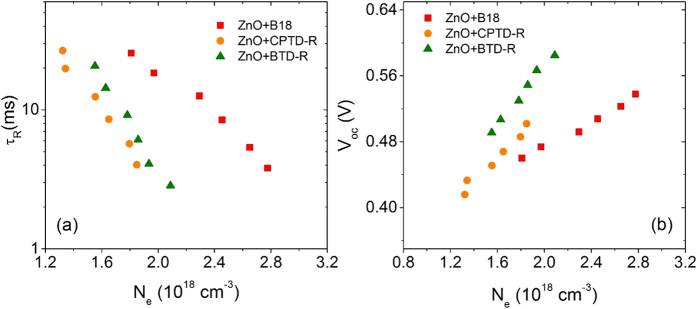
Comparison of electron-transport kinetics for three different dyes sensitized hierarchical ZnO: (**a**) *τ*_R_ vs *N*_e_; (**b**) *V*_oc_ vs *N*_e_; (*τ*_R_: electron lifetime, *V*_oc_: open-circuit voltage, *N*_e_: charge density).

B18 dye has optimized electron injection, fast oxidized dye regeneration reaction and better γ_LHE_ compared to other two dyes (BTD-R and CPTD-R), which contributes to better *J*_sc_, hence enhancing *η*. BTD-R features better photovoltaic performances than CPTD-R dye when applied to hierarchical structured ZnO nanocrystalline, which is opposite to the trend observed in mesoporous standard TiO_2_. In that case, CPTD-R seems featuring better γ_in_ than BTD-R, which is dominant over the oxidized dye regeneration efficiency. This behaviour is mainly attributed to the nature of photoanode material and is consistent with literature, as TiO_2_ features faster electron injection efficiency compared to ZnO^42^,^61^. These results are further supported by IPCE and TCD/TVD measurements in terms of better IPCE values (see Fig. 3(b)) and longer *τ*_R_ (Fig. 6(a)). IPCE values coherently follow the trend observed for *J*_sc_ (i.e B18 > BTD-R > CPTD-R) and overall the spectra show greatly enhanced values as compared with those observed in case of TiO_2_ photoanodes.

An extension of IPCE spectra, as compared with absorption specta recorded for dye solutions (Fig. 2(a)), from 650 to 700 nm is observed in case of both TiO_2_ and ZnO devices. This behavior has been already observed in literature^30^ and can be ascribed to two main reasons. Upon adsorption on metal oxides, conjugation length extension of sensitizers is changed, due to the anchoring on metal oxide by carboxylic groups, which can reflect in IPCE at longer wavelengths. Another reason is the possible J- or H- aggregation of dyes on TiO_2_: the large amount of adsorbed sensitizers may result in head-to-head or/and face-to-face aggregation on the adsorption state, which is also beneficial to extend the IPCE response of devices.

*V*_oc_ values of devices follow the trend B18 > BTD-R > CPTD-R, which appears mainly dependent on charge recombination, as indicated by the *τ*_R_ obtained for the corresponding devices and shown in Fig. 6(a). τR values of respectives devices at particular *N*_e_ (1.8 × 10^18^ cm^−3^) reported in Table S3. The device sensitized with B18 features highest *τ*_R_ and hence possible reduced recombination of the electrons in the CB of the semiconductor with redox couple species of the electrolyte. Sligth differences were observed in the TVD plots (Fig. 6(b), Table S3) and possible shift of the CBE of ZnO upon dye uptake seems negligible, which does not help in enhancing the open circuit voltage of the devices. Similar situation was observed with mesoporous TiO_2_.

Finally, relatively low FFs were found for ZnO-based DSSCs. Lower FF values for ZnO, as compared with TiO_2_, have been often reported by previous studies (an overview on ZnO-based solar cells can be found in ref. 62) and mainly attributed to poor injection of photogenerated charges from the dye to the metal oxide. However, numerical simulation on *J*-*V* characteristics have also pointed out that series resistances can be also partially responsible of the relatively low FFs recorded in ZnO real devices^63^.

### Comparison of hierarchical ZnO nanocrystallites and standard mesoporous TiO_2_

**Figure 7:**
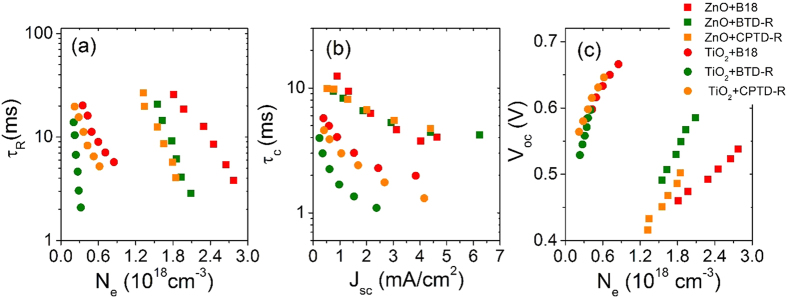
Comparison of electron-transport kinetics for hierarchical structured ZnO and standard mesoporous TiO_2_ sensitized by B18, CPTD-R and BTD-R based DSSCs: (**a**) *τ*_R_ vs *N*_e_; (**b**) *τ*_C_ vs *J*_sc_ and (**c**) *V*_oc_ vs *N*_e_.

A systematic comparison of the current-density vs voltage and IPCE for hierarchical structured ZnO and standard mesoporous TiO_2_ film of comparable thickness sensitized by the three different metal-free organic dyes are displayed in Figure S2 and the corresponding functional parameters are reported in Table S1. *V*_oc_ values are lower for all dyes (20% for B18, 23% for CPTD-R and 11% for BTD-R), when used as light harvesters in ZnO-based DSSCs: TiO_2_-based devices exhibit systematically higher voltage (Fig. 6 (c)), indicating more negative values of CBE for titanium dioxide. As reported by Hara *et al*.^64^, large driving force for back electron transfer (from the metal oxide to the redox couples in the electrolyte) is associated to electrons located in shallow CBE, resulting in short lifetime, which seems confirmed also in our case (Fig. 7 (a)).

In contrast to *V*_oc_, all dyes give higher *J*_sc_ in ZnO than in TiO_2_. This can be explained on the basis of better electron transport properties featured by ZnO (one order of magnitude higher than TiO_2_^30^) and improved light harvesting in hierarchical assembled ZnO, as we previously reported^53^.

### Effect of blocking layer

**Figure 8:**
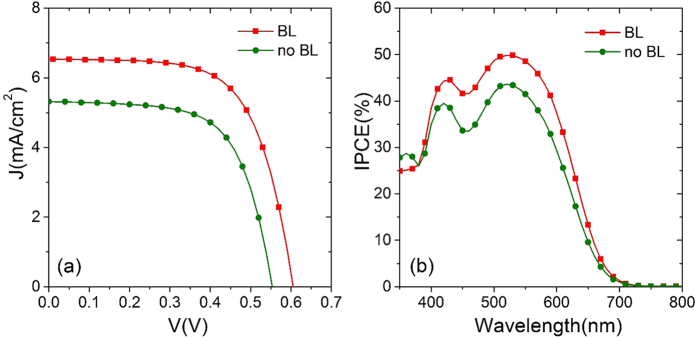
(**a**) Current density vs photovoltage under 1 sun illumination (AM 1.5 G, 100 mW cm^−2^); (**b**) IPCE spectra for hierarchical structured ZnO DSSCs with and without BL sensitized by B18.

We verified the effect of the application of a compact ZnO blocking layer (BL) between FTO and active layer of hierarchical structured ZnO. The beneficial role of BL in boosting *η* in ZnO DSSCs was already demonstrated for the commercial dye N719^53^,^59^. Here, we extend this concept also to hierarchical structured ZnO photoanodes sensitized with B18 dye. Cell provided with a ZnO BL delivered better functional performances as compared with its counterpart without BL (shown in Fig. 8 and corresponding photovoltaic parameters are reported in Table 3).

The highly favorable effect of the BL is clearly visible in *J*-*V* curves (see Fig. 8(a)): *J*_sc_ was indeed enhanced of about 24% and *V*_oc_ increased by 52 mV in the device provided by the compact ZnO BL as compared to the device without BL, which result in an overall enhancement of photoconversion efficiency of almost 35%. In a previous work^59^, investigation through electrochemical impedance spectroscopy demonstrated that the role of BL is especially related to an increased chemical capacitance, resulting in an overall increased *τ*_R_.

Figure 8(b) shows the beneficial effect of the BL in IPCE spectra, too. Increased IPCE for the cell with BL is consistent with the enhancement in *J*_sc_.

**Figure 9:**
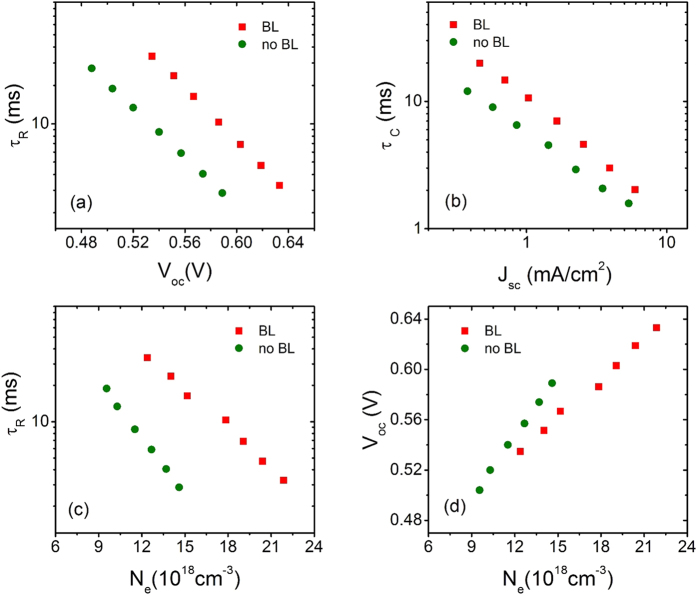
Comparison of electron-transport kinetics for hierarchical structured ZnO DSSCs with and without BL sensitized by B18: (**a**) *τ_R_* vs *V_oc_*; (**b**) *τ_C_* vs *J_sc_* (**c**) *τ_R_* vs *N_e_* and (**d**) *V_oc_* vs *N_e_*.

To investigate more in detail about the effect of BL on electron transport and recombination kinetics, we used temporally resolved techniques TCD and TVD under short-circuit and open-circuit conditions, respectively. Figure 9(a) collects the *τ*_R_ versus *V*_oc_ taken at open circuit condition. The cell with BL has systematically higher *τ*_R_ than cell without BL, when both cells are compared at same *V*_oc_ level (see Table S6). This reflects the beneficial role of BL to reduce the carrier recombination at the FTO/electrolyte interface by physically insulation of the FTO from electrolyte^55^,^56^.

Figure 9(b) shows plot of electron collection time (*τ*_C_) versus current density (*J*_sc_) obtained from charge extraction (CE) data under the same condition as mentioned above. In principle, electron collection time for both cells (with and without BL) should be the same, as the active layer of both the cells is the same. The higher value of *τ*_C_ in the cell with BL than without BL at particular value of *J*_sc_ (see Table S6) is probably due to better physical contact between the active layer of ZnO nanoparticles and the FTO glass substrate^65^. So, the longer electron life time and the improved electron collection efficiency are responsible for higher *J*_sc_ and *V*_oc_ (see Table 3) and hence the conversion efficiency of the device with BL.

Figure 9(c) shows plots of *τ*_R_ versus *N*_e_. The *τ*_R_ for the cell with BL and without BL decreases with increase in charge density. At fixed value of *N*_e_, cell with BL has higher value of *τ*_R_ (see Table S6). These results demonstrated that more charge is accumulated for the cell with BL, which explain the trend of *V*_oc_ with BL > *V*_oc_ no BL.

Figure 9(d) displays the plots of *V*_oc_ versus *N*_e_. The *V*_oc_ for both cells increases with charge density. At fixed value of *N*_e_, the *V*_oc_ of the cell with BL is higher than the *V*_oc_ without BL (see Table S6). Typically, two possible reasons exist for this improvement in *V*_oc_: one is the reduced recombination between the injected electron and oxidized species of the electrolyte as we already explained in previous section (Fig. 9(c)), and the second one is the change in CB edge position of metal oxide with respect to the redox potential of the electrolyte^66^. The second possibility in our case is insignificant as we can see in Fig. 9(d) because there is no drastic shift in CB edge positions for the device with and without BL. So, in our case, reduced recombination, which is easily reflected from improved electron life time and collection time, would be responsible for the larger value of *V*_oc_ in the device with BL.

In order to evaluate the solid demonstration and the reproducibility of BL effects on the device functional parameters, we fabricated two devices per kind (with and without BL), while keeping constant all the other parameters. The results demonstrating the high reproducibility of BL effect and of the device fabrication process are reported in Figure S6, and corresponding functional parameters are reported in Table S6 of the Supporting information.

## Conclusions

In summary, we applied three newly synthesized metal-free organic dyes (B18, CPTD-R and BTD-R) as light harvester in DSSCs and carried out systematic comparison of photovoltaic properties of each dye in hierarchical structured ZnO and in benchmarking standard mesoporous TiO_2_ photoanodes. We demonstrated that B18 dye gives better photovoltaic properties than the other two dyes in both hierarchical structured ZnO and commercial TiO_2_ due to higher potential of electron injection and better light harvesting. The TCD/TVD results demonstrated that device with B18 dye has better *τ*_*R*_ and negligible shift in the CB of dye sensitized photoanodes as compared to CPTD-R and BTD-R dyes based devices.

Each dye results in higher *J*_sc_ in hierarchical structured ZnO than in standard mesoporous TiO_2_, mainly due to better electron transport properties featured by hierarchical structured ZnO nanocrystallites compared to TiO_2_. This behavior is further confirmed by TCD/TVD results, which demonstrated that for each dye, the *τ*_R_ and *τ*_c_ are higher in hierarchical structured ZnO nanocrystallites than in standard mesoporous TiO_2_.

In addition, we applied ZnO compact BL between the active layer and the FTO glass in ZnO photoanodes, demonstrating a significant improvement in device functional performances. This interpretation is confirmed by the results provided by TCD/TVD such as improved *τ*_*R*_ and *τ*_*c*_ for a cell with BL as compared to cell without BL. The results of this work can open a new room for the development of ZnO-based DSSCs by proper combination of well-designed metal-free organic dyes and ZnO nanostructured photoanodes.

## Methods

### Dyes synthesis

**Figure 10:**
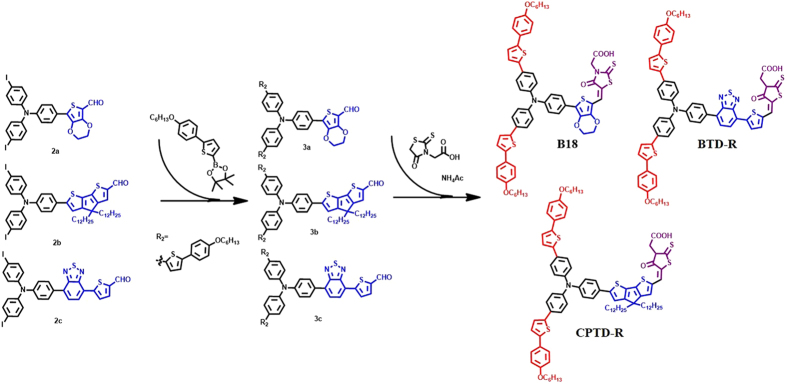
Synthesis of B18, CPTD-R and BTD-R dye molecules.

All solvents and reagents, unless otherwise stated, were of analytical grade quality and used as received. Standard Schlenk techniques were employed to manipulate oxygen- and moisture-sensitive chemicals. 7-(4-(bis(4-iodophenyl)amino)phenyl)-2,3-dihydrothieno[3,4-b][1,4]dioxine-5-carbaldehyde (2a), 6-(4-(bis(4-iodophenyl)amino)phenyl)-4,4-didodecyl-4H-cyclopenta[1,2-b:5,4-b′] dithiophene-2-carbaldehyde (2b), 5-(7-(4-(bis(4-iodophenyl)amino)phenyl)benzo[c][1,2,5]thiadiazol-4-yl)thiophene-2-carbaldehyde (2c) were synthesized according to the literature as shown in figure 10^67^. Tetrahydrofuran (THF) was dried with sodium sand, and benzophenone indicator, dichloromethane (DCM) was dried out with calcium hydride before using. Reactions were carried out under a dry nitrogen atmosphere.

### (E)-2-(5-((7-(4-(bis(4-(5-(4-(hexyloxy)phenyl)thiophen-2-yl)phenyl)amino)phenyl)-2,3-dihydrothieno[3,4-b][1,4]dioxin-5-yl)methylene)-4-oxo-2-thioxothiazolidin-3-yl)acetic acid (B18) synthesis

Compound 2 (93 mg, 0.1 m mol) and rhodanine-3-acetic acid (19 mg, 0.1 m mol), ammonium acetate (3.5 mg, 0.044 m mol) was added into acetic acid (10 ml) under N2. The reaction was stirred at 120 °C for 12 h. The progress of the reaction was monitored with TLC. The solvent was removed under vacuum. The residue was purified on silica chromatograph using DCM/MeOH = 20/1 as eluent. The product was re-crystallized from DCM/MeOH to give red solid of B18 (36 mg, 35%). ^1^H NMR (CDCl_3_) δ H 8.02 (s, 1H), 7.72 (d, J = 8.85 Hz, 2H), 7.24 (d, J = 4.00 Hz, 2H), 7.19 (m, 8H), 6.95 (d, J = 4.87 Hz, 4H), 4.92 (s, 2H), 4.41 (d, J = 6.28 Hz, 4H), 4.00 (t, J = 4.45 Hz, 2H), 1.86 (m, 4H), 1.51 (m, 4H), 1.39 (m, 12H), 0.93 (m, 6H). ^13^C NMR (CDCl3) δ C ppm: 192.0, 166.7, 158.8, 147.4, 146.2, 145.8, 141.9, 137.9, 130.0, 128.0, 127.6, 127.0, 126.9, 126.8, 126.5, 125.7, 125.0,123.4, 123.1, 122.9, 115.6, 115.0, 114.9, 111.0, 68.1, 64.6, 44.4, 31.6, 29.2, 25.7, 22.6, 14.0. IR (KBr, cm^−1^): 3447, 3208, 2927, 2852, 1770, 1705, 1577, 1497, 1477, 1445, 1402, 1364, 1323, 1247, 1179, 1132, 1080, 1022, 937, 831, 797, 698, 635, 585. MS (MALDI-TOF) m/z: calcd for 1103.458; found 1102.360. Element analysis (%) calcd for C_62_H_58_N_2_O_7_S_5_, C, 67.48; H, 5.30; N, 2.54; found C, 67.44, H 5.41; N, 2.60.

### (E)-2-(5-((6-(4-(bis(4-(5-(4-(hexyloxy)phenyl)thiophen-2-yl)phenyl)amino)phenyl)-4,4-didodecyl-4H-cyclopenta[1,2-b:5,4-b′]dithiophen-2-yl)methylene)-4-oxo-2-thioxotetrahydrothiophen-3-yl)acetic acid (CPDT-R) synthesis

The synthesis procedures of CPTD-R dye were according to the literature and it was similar with B18, which is obtained as purple solid. ^1^H NMR (CD_2_Cl_2_) δ H8.01 (s, 1H), 7.60 (m, 10H), 7.34 (s, 1H), 7.28 (d, J = 4.6 Hz, 2H), 7.23 (m, 9H), 6.97 (d, J = 8.2 Hz, 4H), 4.93 (s, 2H), 4.00 (t, J = 4.45 Hz, 2H), 1.86 (m, 4H), 1.51 (m, 4H), 1.39 (m, 12H), 0.93 (m, 6H). ^13^C NMR (CDCl3) δ C ppm: 192.3, 158.8, 147.1, 146.0, 143.3, 142.0, 137.8, 129.7, 128.9, 126.8, 124.6, 122.9, 115.0, 114.9, 78.2, 68.1, 64.6, 44.4, 31.6, 29.2, 25.7, 22.6, 24.6, 14.1;IR (KBr, cm^−1^): 3445, 2927, 2849, 1709, 1572, 1493, 1408, 1322, 1293, 1244, 1205, 1112, 1047, 838, 799, 698, 635, 585; MS (MALDI-TOF) m/z: calcd for 1473.647; found 1472.580. Element analysis (%) calcd for C_90_H_107_NO_5_S_6_, C, 73.28; H, 7.31; N, 0.95; found C, 73.24, H 7.41; N, 0.98.

### (E)-2-(5-((5-(7-(4-(bis(4-(5-(4-(hexyloxy)phenyl)thiophen-2-yl)phenyl)amino)phenyl)benzo[c][1,2,5]thiadiazol-4-yl)thiophen-2-yl)methylene)-4-oxo-2-thioxotetrahydrothiophen-3-yl)acetic acid (BTD-R) synthesis

The synthesis procedures of BTD-R dye were according to the literature and it was similar with B18, which is obtained as red solid. ^1^H NMR (DMSO-d6) δ H8.56 (s, 1H), 8.25 (d, J = 7.7 Hz,2H), 8.04 (d, J = 8.8 Hz, 2H), 7.93 (d, J = 8.2 Hz, 2H), 7.65 (d, J = 9.2 Hz, 4H), 7.57 (d, J = 15.7 Hz, 4H), 7.44 (dd, 4H), 7.23 (d, J = 6.2 Hz, 2H), 7.17 (d, J = 8.3 Hz, 4H), 6.99 (d, J = 9.3 Hz, 4H), 4.91 (s, 2H), 4.00 (t, J = 4.4 Hz, 4H), 1.74 (m, 4H), 1.51 (m, 12H), 0.98 (m, 6H); ^13^C NMR (CDCl3) δ C ppm: 192.2, 168.8, 158.7, 153.7, 14.2, 146.3, 138.4, 135.5, 133.7, 130.3, 126.8, 123.3, 120.9, 114.8, 78.2, 68.1, 64.3, 44.4, 31.6, 29.2, 24.7, 22.6, 24.6, 14.0; IR (KBr, cm^−1^): 3438, 3026, 2927, 2852, 2364, 2338, 1705, 1577, 1497, 1434, 1323, 1247, 1192, 1110, 1057, 845, 785, 698, 635, 585; MS (MALDI-TOF) m/z: calcd for 1177.278; found 1176.772. Element analysis (%) calcd for C_67_H_59_N_3_O_5_S_6_, C, 68.28; H, 5.05; N, 3.57; found C, 68.24, H 5.12; N, 3.63.

### Photoanodes preparation

ZnO BL and active layer were deposited by spray pyrolysis as reported in ref. 53. In brief 0.24 M Zn(CH_3_COO)_2_·2H_2_O (25 mL of methanol/water, 2:1 v/v) is used as precursor for the BL and mixture of ethanolic suspension of commercial ZnO nanoparticles (0.5 g in 15 ml ethanol) and 0.55 M Zn(CH_3_COO)_2_·2H_2_O (40 ml of methanol/water, 3:1 v/v) is used for the active layer. For both the BL and the active layer, the precursors are sprayed using N_2_ carrier gas at pressure of 0.40 bar on FTO glass (sheet resistance 10 Ω/) kept at 250 °C. Nozzle-to-sample distance: 37 cm for the BL, 25 cm for the active layer. Post deposition annealing is carried out at 450 °C for 30 minutes under ambient conditions.

TiO_2_ photoanodes of required thickness were prepared on ultrasonically cleaned FTO glass substrate by repetitive screen printing of a transparent layer (15–20 nm sized) and then a scattering layer (150–200 nm sized). Drying process followed for 10 minutes at ambient atmosphere and temperature and then for 6 minutes at 120 °C. Finally all photoanodes were annealed at 500 °C for 30 minutes under ambient conditions.

Thickness of the both photoanodes (ZnO and TiO_2_) was measured by Dek Tak 150 stylus profiler.

### Pt-counter electrodes

The Pt-counter electrode was prepared by spin-coating a solution containing platinum (H_2_PtCl_6_ in isopropanol) onto the FTO glass at 2000 rpm for 10 s, then heated at 380 °C for 30 minutes.

### Cell fabrication

All photoanodes were treated with ultraviolet ozone (UV-O_3_) cleaning for 18 minutes to remove the surface contaminants, followed by heating at 200 °C for 15 minutes, then let them cool to 80 °C and keeping at 80 °C before immersion in dye solution. For dye uptake, both ZnO and TiO_2_ photoanodes were immersed in 0.2 mM dye solution (dye/Chenode-oxycholic acid (CDCA), 1:1 w/w, in mixture of toluene/ethanol, 4:1 v/v) 3 h for B18 and BTD-R and 2 h for CPTD-Rat room temperature. After dye absorption, the photoanodes were washed first with CH_2_Cl_2_, then with dry ethanol to remove unabsorbed dye molecules and then dried in hot air. DSSCs were fabricated by sealing the dye sensitized photoanodes and Pt-counter electrodes in a sandwich-type structure with a hot-melt film (SX1170, thickness 60 μm) under thermal compression at 90 °C for 10 s. The space between them was filled by redox couple electrolyte, which is composed of 0.5 M PMII; 0.03 M I_2_; 0.5 M TBP in a mixture of acetonitrile and valeronitrile (85:15 v/v).

### Characterizations

^1^H NMR and ^13^CNMR spectra were measured on a Bruker-AF301 AT 400 MHz spectrometer. High resolution mass spectra (HRMS) were measured with a Bruker MALDI TOF mass spectrometer. The UV-visible absorption spectra were observed with a PE950 spectrophotometer and Fluorescent emission spectra were obtained with a Jasco FP-6500 spectrophotometer. FT-IR spectra were recorded on a Bruker VERTEX 70. All cyclic voltammetry measurements were conducted in freshly distilled trichloromethane using TBAPF6 (0.5 M) as supporting electrolyte in a three–electrode system, with each solution being purged with N_2_ before measurement. The working electrode was a Pt disk; the reference electrode was Ag/AgCl and the counter electrode was a Pt rod. All measurements were made at 23 °C with a CHI660C electrochemical work station. The reduction potentials were calibrated with ferrocene as internal reference. The HOMO and LUMO values were transformed according to the literature^68^.

The current-voltage (*I*-*V*) measurements were carried out with a source meter (Keithley 2400, computer-controlled) under one sun simulated sunlight at AM 1.5G (100 mWcm^−2^), calibrated with silicon reference cell. The active area of the cells was 0.25 cm^2^. The incident photon to current conversion efficiency (IPCE) of the devices was measured with a system comprising a Xe lamp (A-1010, PTi, 150 W), monochromator (PTi, 1200 grooves mm^−1^ blazed at 500 nm), and source meter (Keithley 2400). All measurements were carried by using external shadow mask of area 0.5 × 0.5 cm^2^.

Transient photocurrent and photovoltage decay (TCD and TVD) measurements were carried out with a computer-controlled instrumental setup containing two LED light sources. Six steady-state light intensities were obtained as bias irradiations from a white LED on tuning the driving voltage. A green LED (λ = 532 nm) controlled with a pulse generator (DG535, SRS) generated a perturbation pulse of duration 50 ms. Both the pulsed green light and the steady-state white light irradiated the photoanode side of the cell. The pulsed-probe irradiation was controlled with a LED power supply to maintain the modulated photovoltage less than 5 mV in each measurement. The probe beams generated carriers causing a slightly increased photocurrent (Δ*J*_SC_) near *J*_SC_ of the cell at the short-circuit condition, or a slightly increased photovoltage (Δ*V*_OC_) near *V*_OC_ of the cell at the open-circuit condition, subjected to the white bias light; the current and voltage decays were thereby measured, respectively. The resulting photocurrent and photovoltage transients were recorded on a digital oscilloscope (MSO2014, Tektronix); the signals passed a current preamplifier (SR570, SRS) at a short-circuit condition. For the charge extraction method, white light LED is fired under the open-circuit condition for duration 200 ms and is then turned off at the same time that the system is switched to the short-circuit condition.

## Additional Information

**How to cite this article**: Singh Selopal, G. *et al*. Metal-free organic dyes for TiO_2_ and ZnO dye-sensitized solar cells. *Sci. Rep*. **6**, 18756; doi: 10.1038/srep18756 (2016).

## Supplementary Material

Supplementary Information

## Figures and Tables

**Table 1:** Absorption maxima (10^3^/M^−1^ cm^−1^), emission maximums and electrochemical characteristics of B18, CPTD-R and BTD-R dyes.

Dyes	Absorption λ_max_^a^/nm (10^3^/M^−1^ cm^−1^)	Emission^b^ λ_max_/nm	Potentials and energy level		
			E_ox_/V^c^ (vs. NHE)	E_0–0_/V^d^(vs. NHE)	E_ox_–E_0–0_ /V
B18	537(46.4)	677	0.98	2.06	−1.07
CPTD-R	565(59.5)	694	0.77	1.97	−1.20
BTD-R	530(38.7)	715	0.9	2.98	−1.08

^a^Absorption and emission data were measured in CHCl_3_ at 25 °C; Electrochemical measurements were performed at 25 °C with each dye (0.5 mM) in CHCl_3_/0.1 M TBAPF_6_/N_2_, Pt disk working and Pt counter electrodes, Ag/AgCl reference electrode, scan rate = 50 mV s^−1^.

^b^Excitation wavelength/nm: B18, 537; CPTD-R, 565; BTD-R, 530.

^c^First oxidation values.

^d^Estimated from the intersection wavelengths of the normalized UV-vis absorption and the fluorescence spectra.

**Table 2:** Functional performance comparison of three different dyes (B18, CPTD-R and BTD-R) with two metal oxides: Commercial TiO_2_ nanoparticles and hierarchical assembled ZnO nanoparticles based DSSCs.

Photoanode	Dyes	Thickness* (μm)	*V*_oc_ (mV)	*J*_sc_ (mA cm^−2^)	FF (%)	*η* (%)
TiO_2_	B18	16.63	669	5.40	71	2.56
	CPTD-R	17.17	657	3.87	71	1.82
	BTD-R	16.79	590	2.44	68	0.97
ZnO	B18	8.46	543	8.85	56	2.68
	CPTD-R	8.69	504	5.10	56	1.43
	BTD-R	9.87	542	7.14	52	2.03

^*^Total thickness of the ZnO photoanode, including the BL (800 nm thick).

**Table 3:** Effect of BL on the functional performance comparison: B18 dye sensitized hierarchical assembled ZnO nanoparticles based DSSCs with and without the BL.

Photoanode	BL	Thickness* (μm)	V_oc_ (mV)	J_sc_ (mA cm^−2^)	FF (%)	*η* (%)
ZnO	Yes	8.30	609	6.58	64	2.56
	No	9.04	557	5.32	64	1.90

^*^Total thickness of the photoanode, including the BL. 800 nm is the thickness of BL considered in this work.
