# Supplementary material for: Metal-free organic dyes for TiO2 and ZnO dye-sensitized solar cells
Source: Sci Rep. 2016 Jan 7;6:18756. doi: 10.1038/srep18756 (PMC4704050; doi:10.1038/srep18756)
Supplement: Supplementary Information [file srep18756-s1.pdf]

Metal-free organic dyes for TiO_2 and ZnO dye-sensitized solar cells

Supporting Information

Gurpreet Singh Selopal, [1,2] Hui-Ping Wu,[3] Jianfeng Lu,[4] Yu-Cheng Chang,[3], Mingkui Wang,[4] Alberto Vomiero, [2, 5 *] Isabella Concina, [1,2 *] Eric Wei-Guang Diau [3*]

[1] Department of Information Engineering, University of Brescia, Via Valotti 9, 25133 Brescia, Italy.
[2] CNR-INO SENSOR Lab, Via Branze 45, 25123 Brescia, Italy.
[3] Department of Applied Chemistry and Institute of Molecular Science, National Chiao Tung University, Hsinchu 30010, Taiwan.
[4] Michael Grätzel Center for Mesoscopic Solar Cells, Wuhan National Laboratory for Optoelectronics, School of Optical and Electronic Information, Huazhong University of Science and Technology, 1037 Luoyu Road, Wuhan 430074 (P. R. China),
[5] Luleå University of Technology, 971 98 Luleå, Sweden.

* Corresponding authors:
Alberto Vomiero: alberto.vomiero@ltu.se
Isabella Concina: isabella.concina@unibs.it
Eric Diau: diau@mail.nctu.edu.tw

1. Morphological characterization of photoanodes

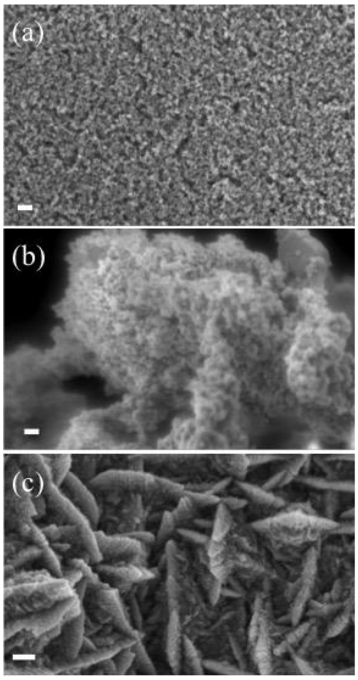

Figure S 1. Scanning electron images of (a) TiO_2 nanoparticles and (b) ZnO hierarchical structures applied as photoanodes. (c) SEM image of ZnO buffer layer. Scale bar: 100 nm.

2. Comparison of hierarchical ZnO nanocrystallites and standard mesoporous TiO_2.

2.1 Dye loading

Figure S2 shows systematic comparison of absorption spectra after the complete removal of dye molecules from the metal oxide (hierarchical structured ZnO and TiO_2) *vs* wavelength (nm).

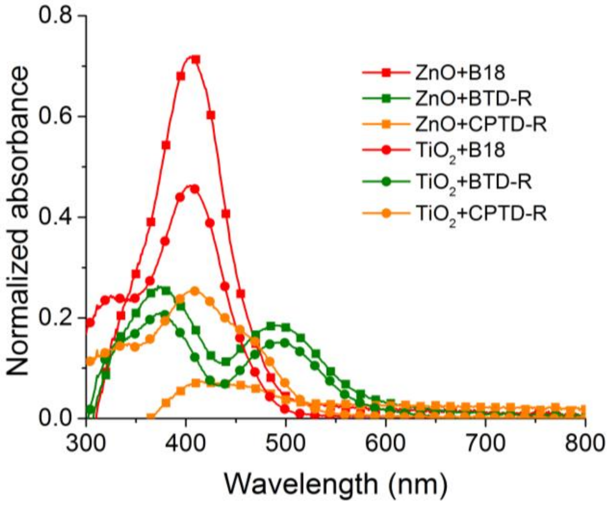

Figure S 2. Comparison of absorption spectra of dye molecules after the complete removal of the dye molecules from the metal oxide: hierarchical structured ZnO (Square) and standard mesoporous TiO_2 (Circle) by B18 (red line), BTD-R (green line) and CPTD-R (orange line).

2.2 J-V and IPCE

Figure S3 shows systematic comparison of the current density vs. photovoltage (*J-V*) curves and IPCE (%) spectra of hierarchical structured ZnO and standard mesoporous TiO_2 photoanodes of comparable thickness sensitized by the three different metal-free organic dyes. Corresponding functional parameters are reported in **Table S1**.

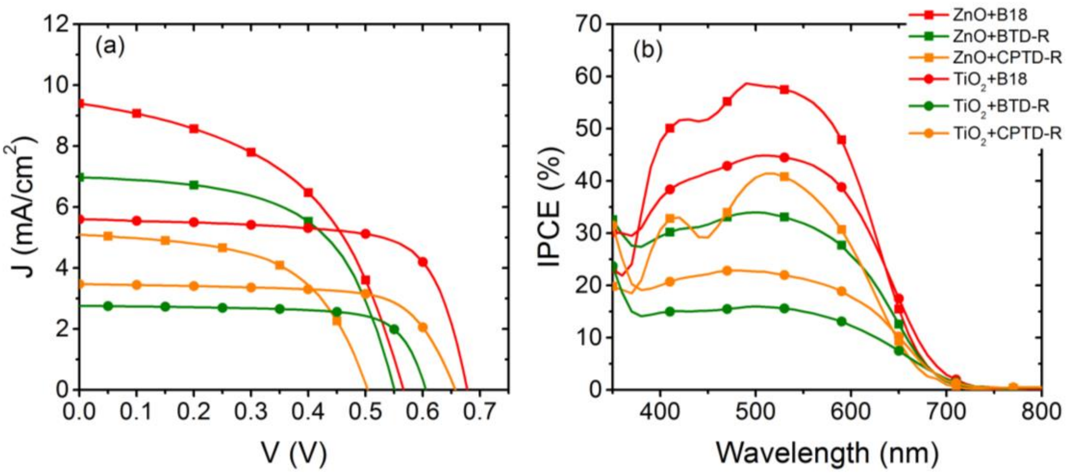

Figure S 3. Comparison of (a) Current density vs photovoltage under 1 sun illumination (AM 1.5G, 100 mW cm^{-2}); (b) IPCE spectra for hierarchical structured ZnO (Square) and standard mesoporous TiO_2 (Circle) based DSSCs sensitized by B18 (red line), BTD-R (green line) and CPTD-R (orange line).

Dye molecule	Metal Oxide	Thickness (μm)	V_{oc} (mV)	J_{sc} (mA cm^{-2})	*FF* (%)	*η* (%)
**B18**	TiO_2	11.07	679	5.60	71	2.69
	ZnO	14.44	544	9.22	54	2.71
**BTD-R**	TiO_2	11.02	607	2.76	73	1.22
	ZnO	13.00	539	7.64	57	2.35
**CPTD-R**	TiO_2	10.81	657	3.47	70	1.60
	ZnO	8.69	504	5.10	56	1.43

Table S 1. Comparison of the functional performances of TiO_2- and ZnO-based DSSCs sensitized with dye molecules B18, CPTD-R and BTD-R under AM1.5G irradiation (100 mW cm^{-2}).

2.3 Transient photocurrent and photovoltage decay measurements

Sample Code	Dye molecules	τ_R *(ms)* (at 3.0×10^{18}cm^{-3})	V_{oc} *(V)* (at 3.0×10^{18}cm^{-3})
T12	B18	24.95	0.574
T3	BTD-R	3.03	0.578
T15	CPTD-R	15.58	0.580

Table S 2. Comparison of the functional properties of TiO_2 based DSSCs sensitized with B18, BTD-R and CPTD-R calculated from transient photocurrent and photovoltage decay (TCD/TVD).

Sample Code	Dye molecules	τ_R (ms) (at 1.8×10^{18}cm^{-3})	V_{oc} (V) (at 1.8×10^{18}cm^{-3})
B22	B18	25.53	0.459
B10	BTD-R	9.10	0.531
C17	CPTD-R	5.70	0.487

Table S 3. Comparison of the functional properties of ZnO- based DSSCs sensitized with B18, BTD-R and CPTD-R calculated from transient photocurrent and photovoltage decay (TCD/TVD).

3. Effect of active layer thickness

The effect of thickness of active layer on the functional properties of all DSSCs, was investigated. Systematic comparisons of the functional performances are shown in **Figure S4** for TiO_2 photoanodes and in **Figure S5** for ZnO photoanodes. Corresponding functional parameters are reported in Table S2 and Table S3, respectively.

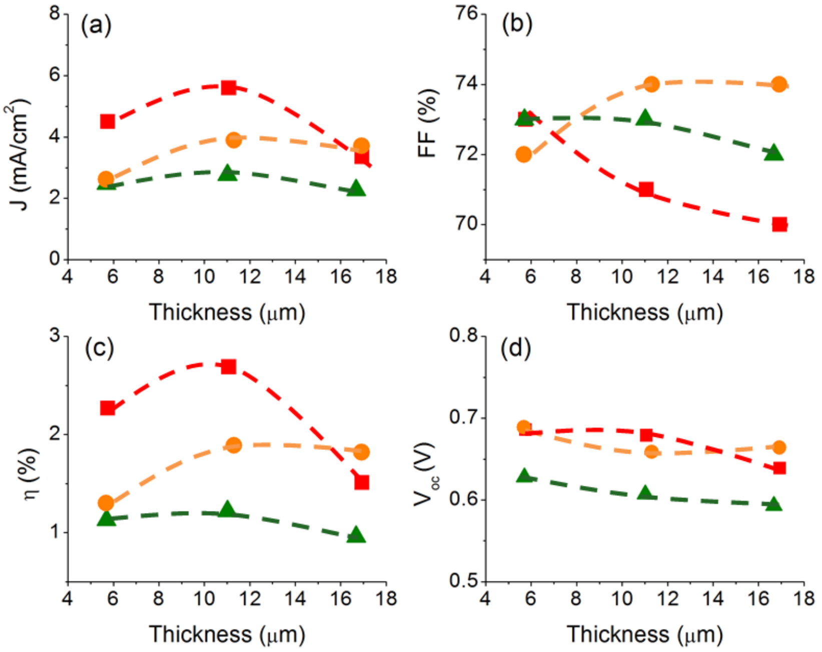

Figure S 4. Functional properties comparison of DSSCs based on standard mesoporous TiO_2 film sensitized with three different dye molecules (Red square= B18, Green triangle= BTD-R and Orange circle= CPTD-R) under AM1.5G irradiation (100 mW cm^{-2}) as a function of photoanode thickness.

Dye molecules	Thickness* (μm)	V_{oc} (mV)	J_{sc} (mA cm^{-2})	*FF* (%)	*η* (%)
**B18**	5.75	686	4.50	73	2.27
	11.07	679	5.60	71	2.69
	16.93	639	3.36	70	1.51
**BTD-R**	5.70	628	2.47	73	1.13
	11.02	607	2.76	73	1.22
	16.68	593	2.27	72	0.96
**CPTD-R**	5.68	689	2.62	72	1.30
	11.30	659	3.89	74	1.89
	16.91	664	3.71	74	1.82

Table S 4. Functional properties of standard mesoporous TiO_2 photoanodes sensitized with B18, BTD-R and CPTD-R under AM1.5G irradiation (100 mW cm^{-2}) as a function of photoanode thickness.

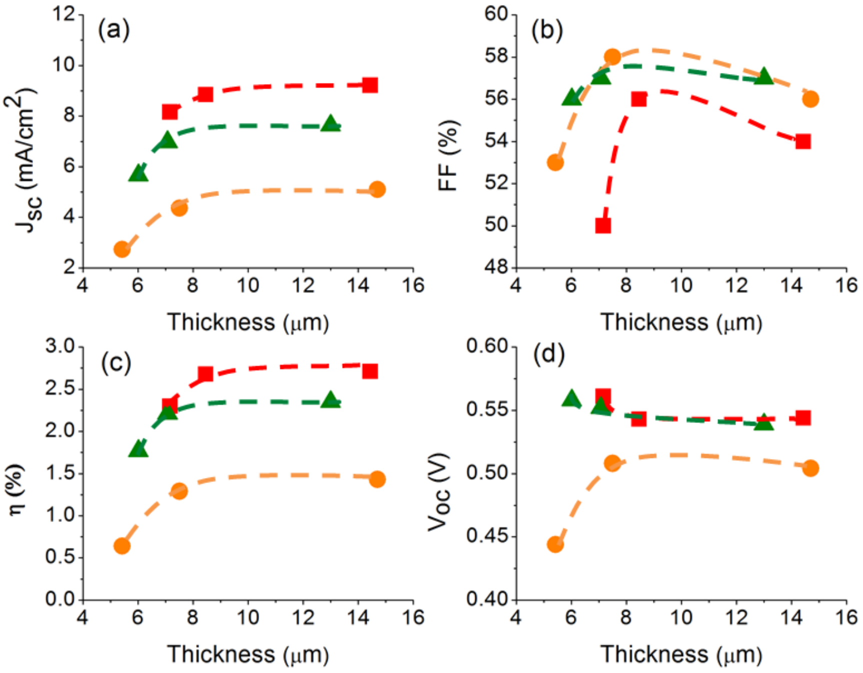

Figure S 5. Functional properties comparison of DSSCs based on hierarchical assembled ZnO nanostructures sensitized with three different dye molecules (Red square= B18, Green triangle= BTD-R and Orange circle= CPTD-R) under AM1.5G irradiation (100 mW cm^{-2}) as a function of photoanode thickness

**Dye molecules**	**Thickness (μm)**	V_{oc} **(mV)**	J_{sc} **(mA cm^{-2})**	***FF*** **(%)**	η **(%)**
B18	7.16	561	8.16	50	2.30
	8.46	543	8.85	56	2.68
	14.44	544	9.22	54	2.71
BTD-R	6.01	558	5.67	56	1.77
	7.07	552	6.98	57	2.21
	13.00	539	7.64	57	2.35
CPTD-R	5.42	444	2.73	53	0.64
	7.50	508	4.36	58	1.29

14.70	504	5.10	56	1.43

Table S 5. Functional properties of hierarchical assembled ZnO nanostructured photoanodes sensitized with B18, BTD-R and CPTD-R under AM1.5G irradiation (100 mW cm^{-2}) as a function of photoanode thickness.

4. Reproducibility of the effect of blocking layer

Functional performances of hierarchically self-assembled ZnO nanocrystallites photoanodes with and without ZnO blocking layer (BL) are presented in **Figure S6** and **Table S6**.

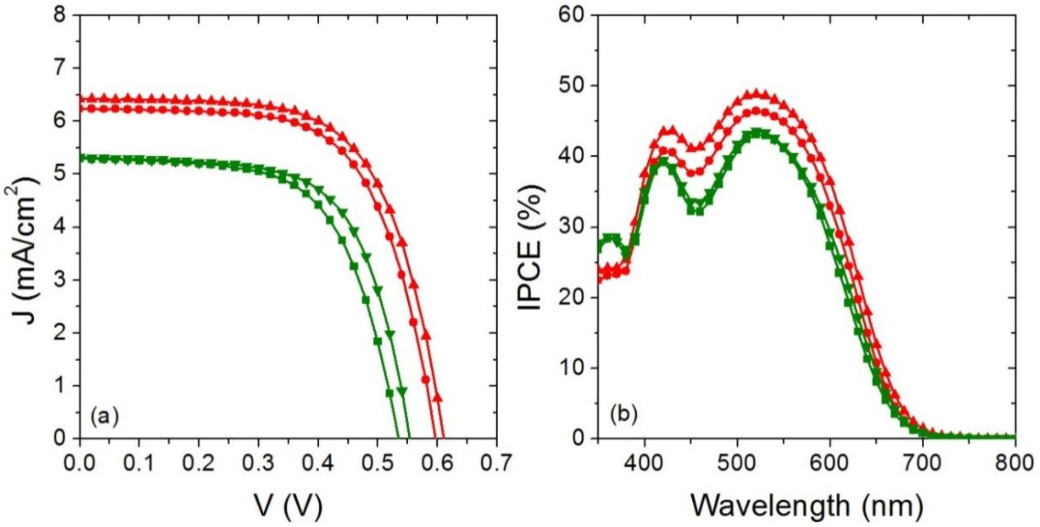

Figure S 6. Comparison of (a) current density vs photovoltage curves; (b) IPCE spectra, for DSSCs with and without blocking layer between FTO and active layer (red line: with BL; green line: without BL).

Sample Code	BL	Thickness* (μm)	V_{oc} (mV)	J_{sc} (mA cm^{-2})	*FF* (%)	η (%)
A6	Yes	8.31	613	6.43	64	2.52
A7	Yes	8.16	601	6.24	64	2.39
B11	No	9.04	557	5.32	64	1.90
C8	No	12.46	538	5.30	62	1.77

*Thickness includes BL (as thick as 0.8 μm)

Table S 6. Functional properties of ZnO-based DSSCs with and without blocking layer

**Sample Code**	**BL**	**τ_R (ms) (at 0.56 V)**	**τ_R (ms) (at 1 mA cm^{-2})**	**τ_R (ms) (at 13.5×10^{18}cm^{-3})**	**V_{oc} (V) (at 13.5×10^{18}cm^{-3})**
A5	Yes	20.41	11.00	27.93	0.548
B11	No	5.57	6.00	4.23	0.568

Table S 7. Comparison of functional properties of B18 sensitized hierarchical assembled ZnO nanostructured based DSSCs with and without blocking layer calculated by transient photocurrent and photovoltage decay (TCD/TVD)
